# 5-Fluoro-3-(4-fluoro­phenyl­sulfon­yl)-2-methyl-1-benzofuran

**DOI:** 10.1107/S1600536810025468

**Published:** 2010-07-03

**Authors:** Hong Dae Choi, Pil Ja Seo, Byeng Wha Son, Uk Lee

**Affiliations:** a Department of Chemistry, Dongeui University, San 24 Kaya-dong Busanjin-gu, Busan 614-714, Republic of Korea; b Department of Chemistry, Pukyong National University, 599-1 Daeyeon 3-dong, Nam-gu, Busan 608-737, Republic of Korea

## Abstract

In the title compound, C_15_H_10_F_2_O_3_S, the 4-fluoro­phenyl ring makes a dihedral angle of 73.20 (4)° with the plane of the benzofuran fragment. The crystal structure is stabilized by aromatic π–π inter­actions between the furan and benzene rings of neighbouring mol­ecules [centroid–centroid distance = 3.805 (3) Å]. The crystal structure also exhibits weak inter­molecular C—H⋯O and C—H⋯F inter­actions.

## Related literature

For the pharmacological activity of benzofuran compounds, see: Aslam *et al.* (2006 ▶); Galal *et al.* (2009 ▶); Khan *et al.* (2005 ▶). For natural products with benzofuran rings, see: Akgul & Anil (2003 ▶); Soekamto *et al.* (2003 ▶). For the structures of related 5-halo-2-methyl-3-phenyl­sulfonyl-1-benzofuran derivatives, see: Choi *et al.* (2008**a* ▶,*b* ▶,c* ▶).
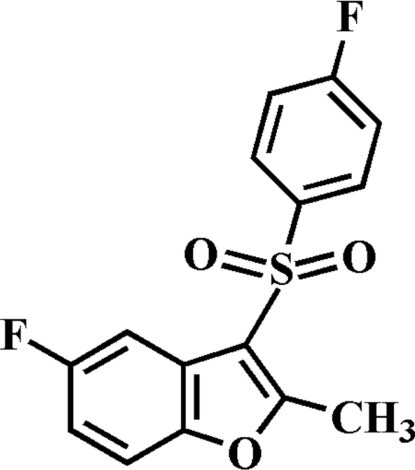


## Experimental

### 

#### Crystal data

C_15_H_10_F_2_O_3_S
*M* *_r_* = 308.29
Triclinic, 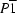

*a* = 7.2799 (9) Å
*b* = 9.5161 (12) Å
*c* = 10.1052 (13) Å
α = 89.844 (2)°
β = 75.057 (2)°
γ = 74.558 (2)°
*V* = 650.32 (14) Å^3^
*Z* = 2
Mo *K*α radiation
μ = 0.28 mm^−1^
*T* = 173 K
0.40 × 0.40 × 0.30 mm

#### Data collection

Bruker SMART APEXII CCD diffractometer
Absorption correction: multi-scan (*SADABS*; Bruker, 2009 ▶) *T*_min_ = 0.674, *T*_max_ = 0.746
5657 measured reflections
2797 independent reflections
2404 reflections with *I* > 2σ(*I*)
*R*_int_ = 0.017

#### Refinement

*R*[*F* ^2^ > 2σ(*F* ^2^)] = 0.034
*wR*(*F* ^2^) = 0.092
*S* = 1.10
2797 reflections
191 parameters
H-atom parameters constrained
Δρ_max_ = 0.25 e Å^−3^
Δρ_min_ = −0.39 e Å^−3^

### 

Data collection: *APEX2* (Bruker, 2009 ▶); cell refinement: *SAINT* (Bruker, 2009 ▶); data reduction: *SAINT*; program(s) used to solve structure: *SHELXS97* (Sheldrick, 2008 ▶); program(s) used to refine structure: *SHELXL97* (Sheldrick, 2008 ▶); molecular graphics: *ORTEP-3* (Farrugia, 1997 ▶) and *DIAMOND* (Brandenburg, 1998 ▶); software used to prepare material for publication: *SHELXL97*.

## Supplementary Material

Crystal structure: contains datablocks global, I. DOI: 10.1107/S1600536810025468/zl2288sup1.cif

Structure factors: contains datablocks I. DOI: 10.1107/S1600536810025468/zl2288Isup2.hkl

Additional supplementary materials:  crystallographic information; 3D view; checkCIF report

## Figures and Tables

**Table 1:** Hydrogen-bond geometry (Å, °)

*D*—H⋯*A*	*D*—H	H⋯*A*	*D*⋯*A*	*D*—H⋯*A*
C11—H11⋯O2^i^	0.93	2.45	3.247 (2)	144
C14—H14⋯F2^ii^	0.93	2.53	3.441 (2)	168

Symmetry codes: (i) 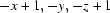; (ii) 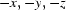.

**Fig. 1.**
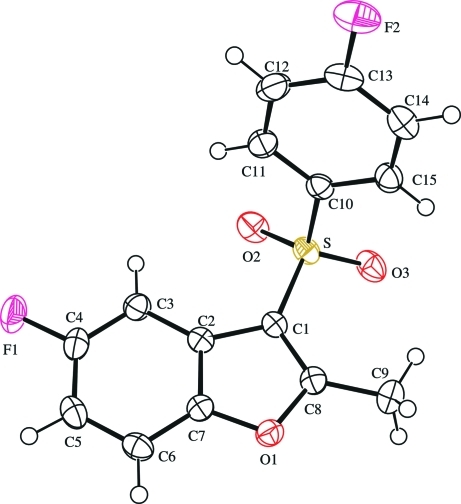
The molecular structure of the title compound with the atom numbering scheme. Displacement ellipsoids are drawn at the 50% probability level. H atoms are presented as a small spheres of arbitrary radius.

**Fig. 2.**
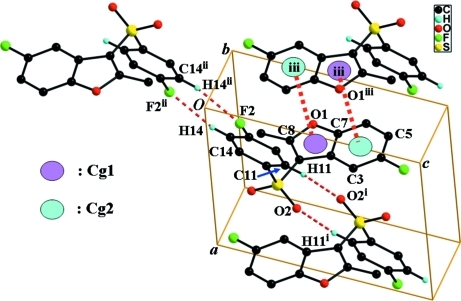
π–π, C—H···O, and C—H···F interactions (dotted lines) in the crystal structure of the title compound. Cg1 and Cg2 denote the ring centroids. [Symmetry codes: (i) - x + 1, - y, - z + 1; (ii) - x, - y, - z; (iii) - x, - y + 1, -z + 1.]

## supplementary crystallographic information

### Comment

Compounds containing a benzofuran skeleton show diverse pharmacological properties such as antifungal (Aslam *et al.*, 2006), antitumor and antiviral (Galal *et al.*, 2009), and antimicrobial (Khan *et al.*, 2005) activities. These compounds widely occur in nature (Akgul & Anil, 2003; Soekamto *et al.*, 2003). As a part of our study of the substituent effect on the solid state structures of 5-halo-2-methyl-3-phenylsulfonyl-1-benzofuran analogues (Choi *et al.*, 2008*a,b,c*), we report the crystal structure of the title compound (Fig. 1).

The benzofuran unit is essentially planar, with a mean deviation of 0.013 (1) Å from the least-squares plane defined by the nine constituent atoms. The dihedral angle formed by the benzofuran plane and the 4-fluorophenyl ring is 73.20 (4)°. The crystal packing (Fig. 2) is stabilized by aromatic π–π interactions between the furan and the benzene rings of neighbouring molecules, with a Cg1···Cg2^iii^ distance of 3.805 (3) Å (Cg1 and Cg2 are the centroids of the C1/C2/C7/O1/C8 furan ring and the C2–C7 benzene ring, respectively). The molecular packing (Fig. 2) is further stabilized by a weak intermolecular C—H···O hydrogen bond between the 4-fluorophenyl H atom and the oxygen of the O═S═O unit (C11—H11···O2^i^, see Table 1 for symmetry operator and numerical values). The crystal packing (Fig. 2) also exhibits C—H···F hydrogen bonds between the 4-fluorophenyl H atom and the fluorine of 4-fluorophenyl ring (C14—H14···F2^ii^, Table 1).

### Experimental

77% 3-chloroperoxybenzoic acid (381 mg, 1.7 mmol) was added in small portions to a stirred solution of 5-fluoro-3-(4-fluorophenylsulfanyl)-2-methyl-1-benzofuran (221 mg, 0.8 mmol) in dichloromethane (40 mL) at 273 K. After being stirred at room temperature for 8h, the mixture was washed with saturated sodium bicarbonate solution and the organic layer was separated, dried over magnesium sulfate, filtered and concentrated at reduced pressure. The residue was purified by column chromatography (benzene) to afford the title compound as a colorless solid [yield 76%, m.p. 426–427 K; *R*_f_ = 0.49 (benzene)]. Single crystals suitable for X-ray diffraction were prepared by slow evaporation of a solution of the title compound in benzene at room temperature.

### Refinement

All H atoms were positioned geometrically and refined using a riding model, with C—H = 0.93 Å for aryl and 0.96 Å for methyl H atoms. *U*_iso_(H) = 1.2*U*_eq_(C) for aryl and 1.5*U*_eq_(C) for methyl H atoms.

### Crystal data

**Table:**

C_15_H_10_F_2_O_3_S	*Z* = 2
*M**_r_* = 308.29	*F*(000) = 316
Triclinic, *P*1	*D*_x_ = 1.574 Mg m^−^^3^
Hall symbol: -P 1	Mo *K*α radiation, λ = 0.71073 Å
*a* = 7.2799 (9) Å	Cell parameters from 3365 reflections
*b* = 9.5161 (12) Å	θ = 2.9–27.5°
*c* = 10.1052 (13) Å	µ = 0.28 mm^−^^1^
α = 89.844 (2)°	*T* = 173 K
β = 75.057 (2)°	Block, colourless
γ = 74.558 (2)°	0.40 × 0.40 × 0.30 mm
*V* = 650.32 (14) Å^3^	

### Data collection

**Table:**

Bruker SMART APEXII CCD diffractometer	2797 independent reflections
Radiation source: rotating anode	2404 reflections with *I* > 2σ(*I*)
graphite multilayer	*R*_int_ = 0.017
Detector resolution: 10.0 pixels mm^-1^	θ_max_ = 27.0°, θ_min_ = 2.1°
φ and ω scans	*h* = −9→9
Absorption correction: multi-scan (*SADABS*; Bruker, 2009)	*k* = −12→12
*T*_min_ = 0.674, *T*_max_ = 0.746	*l* = −12→12
5657 measured reflections	

### Refinement

**Table:**

Refinement on *F*^2^	Primary atom site location: structure-invariant direct methods
Least-squares matrix: full	Secondary atom site location: difference Fourier map
*R*[*F*^2^ > 2σ(*F*^2^)] = 0.034	Hydrogen site location: difference Fourier map
*wR*(*F*^2^) = 0.092	H-atom parameters constrained
*S* = 1.10	*w* = 1/[σ^2^(*F*_o_^2^) + (0.0387*P*)^2^ + 0.2972*P*] where *P* = (*F*_o_^2^ + 2*F*_c_^2^)/3
2797 reflections	(Δ/σ)_max_ < 0.001
191 parameters	Δρ_max_ = 0.25 e Å^−^^3^
0 restraints	Δρ_min_ = −0.39 e Å^−^^3^

### Special details

**Table:**

Geometry. All esds (except the esd in the dihedral angle between two l.s. planes) are estimated using the full covariance matrix. The cell esds are taken into account individually in the estimation of esds in distances, angles and torsion angles; correlations between esds in cell parameters are only used when they are defined by crystal symmetry. An approximate (isotropic) treatment of cell esds is used for estimating esds involving l.s. planes.
Refinement. Refinement of F^2^ against ALL reflections. The weighted R-factor wR and goodness of fit S are based on F^2^, conventional R-factors R are based on F, with F set to zero for negative F^2^. The threshold expression of F^2^ > 2sigma(F^2^) is used only for calculating R-factors(gt) etc. and is not relevant to the choice of reflections for refinement. R-factors based on F^2^ are statistically about twice as large as those based on F, and R- factors based on ALL data will be even larger.

### Fractional atomic coordinates and isotropic or equivalent isotropic displacement parameters (Å^2^)

**Table:**

	*x*	*y*	*z*	*U*_iso_*/*U*_eq_	
S	0.53634 (6)	0.20772 (5)	0.28012 (4)	0.02732 (13)	
F1	0.23174 (19)	0.31294 (13)	0.87101 (11)	0.0452 (3)	
F2	−0.00277 (18)	−0.11034 (13)	0.17822 (13)	0.0504 (3)	
O1	0.20792 (17)	0.60640 (12)	0.41364 (12)	0.0278 (3)	
O2	0.63798 (19)	0.12784 (14)	0.37331 (13)	0.0368 (3)	
O3	0.64674 (18)	0.23753 (14)	0.14903 (13)	0.0370 (3)	
C1	0.3867 (2)	0.37201 (17)	0.36913 (16)	0.0241 (3)	
C2	0.3083 (2)	0.39702 (17)	0.51670 (16)	0.0237 (3)	
C3	0.3194 (3)	0.31470 (19)	0.63059 (17)	0.0282 (4)	
H3	0.3883	0.2162	0.6217	0.034*	
C4	0.2220 (3)	0.3886 (2)	0.75675 (17)	0.0313 (4)	
C5	0.1178 (3)	0.5354 (2)	0.77703 (18)	0.0315 (4)	
H5	0.0569	0.5787	0.8654	0.038*	
C6	0.1057 (2)	0.61649 (19)	0.66459 (18)	0.0295 (4)	
H6	0.0370	0.7151	0.6742	0.035*	
C7	0.2007 (2)	0.54383 (17)	0.53726 (17)	0.0240 (3)	
C8	0.3217 (2)	0.50019 (18)	0.31296 (17)	0.0262 (3)	
C9	0.3471 (3)	0.5460 (2)	0.17131 (18)	0.0368 (4)	
H9A	0.4197	0.4629	0.1077	0.055*	
H9B	0.4185	0.6187	0.1588	0.055*	
H9C	0.2197	0.5861	0.1552	0.055*	
C10	0.3712 (2)	0.11336 (17)	0.25046 (17)	0.0255 (3)	
C11	0.2913 (3)	0.03091 (18)	0.35213 (18)	0.0318 (4)	
H11	0.3231	0.0266	0.4357	0.038*	
C12	0.1637 (3)	−0.04468 (19)	0.3270 (2)	0.0365 (4)	
H12	0.1094	−0.1012	0.3929	0.044*	
C13	0.1192 (3)	−0.03435 (19)	0.2028 (2)	0.0353 (4)	
C14	0.1938 (3)	0.0481 (2)	0.10132 (19)	0.0349 (4)	
H14	0.1589	0.0535	0.0188	0.042*	
C15	0.3225 (3)	0.12286 (19)	0.12576 (17)	0.0299 (4)	
H15	0.3760	0.1791	0.0591	0.036*	

### Atomic displacement parameters (Å^2^)

**Table:**

	*U*^11^	*U*^22^	*U*^33^	*U*^12^	*U*^13^	*U*^23^
S	0.0230 (2)	0.0282 (2)	0.0273 (2)	−0.00234 (16)	−0.00558 (16)	−0.00427 (16)
F1	0.0603 (8)	0.0539 (7)	0.0261 (5)	−0.0203 (6)	−0.0153 (5)	0.0133 (5)
F2	0.0482 (7)	0.0426 (7)	0.0666 (8)	−0.0219 (6)	−0.0165 (6)	−0.0066 (6)
O1	0.0291 (6)	0.0244 (6)	0.0282 (6)	−0.0056 (5)	−0.0066 (5)	0.0038 (5)
O2	0.0312 (7)	0.0346 (7)	0.0412 (7)	0.0030 (5)	−0.0159 (6)	−0.0035 (6)
O3	0.0317 (7)	0.0438 (8)	0.0311 (7)	−0.0125 (6)	0.0014 (5)	−0.0081 (6)
C1	0.0227 (8)	0.0255 (8)	0.0235 (8)	−0.0062 (6)	−0.0058 (6)	−0.0006 (6)
C2	0.0209 (7)	0.0253 (8)	0.0257 (8)	−0.0071 (6)	−0.0070 (6)	0.0002 (6)
C3	0.0299 (9)	0.0266 (8)	0.0287 (9)	−0.0060 (7)	−0.0107 (7)	0.0035 (7)
C4	0.0338 (9)	0.0404 (10)	0.0242 (8)	−0.0162 (8)	−0.0095 (7)	0.0077 (7)
C5	0.0289 (9)	0.0405 (10)	0.0240 (8)	−0.0122 (7)	−0.0020 (7)	−0.0047 (7)
C6	0.0245 (8)	0.0281 (9)	0.0328 (9)	−0.0057 (7)	−0.0037 (7)	−0.0049 (7)
C7	0.0217 (8)	0.0250 (8)	0.0260 (8)	−0.0078 (6)	−0.0061 (6)	0.0028 (6)
C8	0.0246 (8)	0.0291 (8)	0.0256 (8)	−0.0091 (6)	−0.0060 (6)	0.0017 (6)
C9	0.0422 (11)	0.0401 (10)	0.0287 (9)	−0.0124 (8)	−0.0097 (8)	0.0092 (8)
C10	0.0240 (8)	0.0216 (8)	0.0271 (8)	−0.0009 (6)	−0.0058 (6)	−0.0023 (6)
C11	0.0357 (9)	0.0254 (8)	0.0322 (9)	−0.0031 (7)	−0.0112 (8)	0.0030 (7)
C12	0.0412 (11)	0.0247 (9)	0.0415 (10)	−0.0093 (8)	−0.0072 (8)	0.0063 (7)
C13	0.0318 (9)	0.0254 (9)	0.0478 (11)	−0.0068 (7)	−0.0101 (8)	−0.0070 (8)
C14	0.0346 (10)	0.0381 (10)	0.0314 (9)	−0.0072 (8)	−0.0106 (8)	−0.0053 (8)
C15	0.0302 (9)	0.0302 (9)	0.0259 (8)	−0.0055 (7)	−0.0045 (7)	−0.0008 (7)

### Geometric parameters (Å, °)

**Table:**

S—O3	1.4323 (13)	C6—C7	1.380 (2)
S—O2	1.4381 (13)	C6—H6	0.9300
S—C1	1.7422 (16)	C8—C9	1.475 (2)
S—C10	1.7618 (17)	C9—H9A	0.9600
F1—C4	1.3665 (19)	C9—H9B	0.9600
F2—C13	1.352 (2)	C9—H9C	0.9600
O1—C8	1.367 (2)	C10—C15	1.391 (2)
O1—C7	1.3771 (19)	C10—C11	1.393 (2)
C1—C8	1.364 (2)	C11—C12	1.387 (3)
C1—C2	1.448 (2)	C11—H11	0.9300
C2—C7	1.394 (2)	C12—C13	1.372 (3)
C2—C3	1.398 (2)	C12—H12	0.9300
C3—C4	1.377 (2)	C13—C14	1.378 (3)
C3—H3	0.9300	C14—C15	1.385 (2)
C4—C5	1.388 (3)	C14—H14	0.9300
C5—C6	1.380 (2)	C15—H15	0.9300
C5—H5	0.9300		
			
O3—S—O2	119.98 (8)	C1—C8—O1	110.39 (14)
O3—S—C1	109.12 (8)	C1—C8—C9	134.23 (16)
O2—S—C1	106.45 (8)	O1—C8—C9	115.37 (15)
O3—S—C10	107.42 (8)	C8—C9—H9A	109.5
O2—S—C10	107.79 (8)	C8—C9—H9B	109.5
C1—S—C10	105.15 (7)	H9A—C9—H9B	109.5
C8—O1—C7	107.03 (12)	C8—C9—H9C	109.5
C8—C1—C2	107.54 (14)	H9A—C9—H9C	109.5
C8—C1—S	126.42 (13)	H9B—C9—H9C	109.5
C2—C1—S	126.04 (12)	C15—C10—C11	121.13 (16)
C7—C2—C3	119.16 (15)	C15—C10—S	119.32 (13)
C7—C2—C1	104.38 (14)	C11—C10—S	119.55 (13)
C3—C2—C1	136.45 (15)	C12—C11—C10	119.08 (17)
C4—C3—C2	115.79 (16)	C12—C11—H11	120.5
C4—C3—H3	122.1	C10—C11—H11	120.5
C2—C3—H3	122.1	C13—C12—C11	118.63 (17)
F1—C4—C3	117.75 (16)	C13—C12—H12	120.7
F1—C4—C5	117.29 (16)	C11—C12—H12	120.7
C3—C4—C5	124.94 (16)	F2—C13—C12	118.23 (17)
C6—C5—C4	119.29 (16)	F2—C13—C14	118.35 (17)
C6—C5—H5	120.4	C12—C13—C14	123.42 (17)
C4—C5—H5	120.4	C13—C14—C15	118.08 (17)
C7—C6—C5	116.58 (16)	C13—C14—H14	121.0
C7—C6—H6	121.7	C15—C14—H14	121.0
C5—C6—H6	121.7	C14—C15—C10	119.64 (17)
O1—C7—C6	125.09 (15)	C14—C15—H15	120.2
O1—C7—C2	110.65 (14)	C10—C15—H15	120.2
C6—C7—C2	124.23 (16)		
			
O3—S—C1—C8	23.50 (17)	C1—C2—C7—C6	−177.47 (15)
O2—S—C1—C8	154.33 (15)	C2—C1—C8—O1	0.33 (18)
C10—S—C1—C8	−91.47 (16)	S—C1—C8—O1	−179.79 (11)
O3—S—C1—C2	−156.64 (14)	C2—C1—C8—C9	−179.30 (18)
O2—S—C1—C2	−25.81 (16)	S—C1—C8—C9	0.6 (3)
C10—S—C1—C2	88.39 (15)	C7—O1—C8—C1	0.06 (18)
C8—C1—C2—C7	−0.58 (18)	C7—O1—C8—C9	179.77 (14)
S—C1—C2—C7	179.54 (12)	O3—S—C10—C15	−20.43 (15)
C8—C1—C2—C3	−179.31 (18)	O2—S—C10—C15	−151.02 (13)
S—C1—C2—C3	0.8 (3)	C1—S—C10—C15	95.72 (14)
C7—C2—C3—C4	−0.7 (2)	O3—S—C10—C11	159.71 (13)
C1—C2—C3—C4	177.87 (17)	O2—S—C10—C11	29.12 (15)
C2—C3—C4—F1	−178.93 (14)	C1—S—C10—C11	−84.14 (14)
C2—C3—C4—C5	−0.4 (3)	C15—C10—C11—C12	1.1 (2)
F1—C4—C5—C6	179.38 (15)	S—C10—C11—C12	−179.07 (13)
C3—C4—C5—C6	0.9 (3)	C10—C11—C12—C13	−0.6 (3)
C4—C5—C6—C7	−0.1 (2)	C11—C12—C13—F2	178.91 (15)
C8—O1—C7—C6	177.63 (15)	C11—C12—C13—C14	−0.4 (3)
C8—O1—C7—C2	−0.46 (17)	F2—C13—C14—C15	−178.45 (15)
C5—C6—C7—O1	−178.90 (15)	C12—C13—C14—C15	0.8 (3)
C5—C6—C7—C2	−1.1 (3)	C13—C14—C15—C10	−0.3 (3)
C3—C2—C7—O1	179.63 (14)	C11—C10—C15—C14	−0.6 (2)
C1—C2—C7—O1	0.64 (17)	S—C10—C15—C14	179.53 (13)
C3—C2—C7—C6	1.5 (2)		

### Hydrogen-bond geometry (Å, °)

**Table:**

*D*—H···*A*	*D*—H	H···*A*	*D*···*A*	*D*—H···*A*
C11—H11···O2^i^	0.93	2.45	3.247 (2)	144
C14—H14···F2^ii^	0.93	2.53	3.441 (2)	168

Symmetry codes: (i) −*x*+1, −*y*, −*z*+1; (ii) −*x*, −*y*, −*z*.
